# A One-Step Electrochemical Aptasensor Based on Signal Amplification of Metallo Nanoenzyme Particles for Vascular Endothelial Growth Factor

**DOI:** 10.3389/fbioe.2022.850412

**Published:** 2022-05-09

**Authors:** ChenYang Mei, Yuanyuan Zhang, Luting Pan, Bin Dong, Xingwei Chen, Qingyi Gao, Hang Xu, Wenjin Xu, Hui Fang, Siyu Liu, Colm McAlinden, Eleftherios I. Paschalis, Qinmei Wang, Mei Yang, Jinhai Huang, A-Yong Yu

**Affiliations:** ^1^ Eye Hospital and School of Ophthalmology and Optometry, Wenzhou Medical University, Wenzhou, China; ^2^ Department of Ophthalmology, Singleton Hospital, Swansea Bay University Health Board, Swansea, United Kingdom; ^3^ Harvard Medical School, Boston, MA, United States; ^4^ Disruptive Technology Laboratory (D.T.L.), Massachusetts Eye and Ear, Department of Ophthalmology, Harvard Medical School, Boston, MA, United States; ^5^ Eye Institute and Department of Ophthalmology, Eye & ENT Hospital, Fudan University, Shanghai, China; ^6^ NHC Key Laboratory of Myopia (Fudan University), Key Laboratory of Myopia, Chinese Academy of Medical Sciences, Shanghai, China; ^7^ Shanghai Research Center of Ophthalmology and Optometry, Shanghai, China

**Keywords:** electrochemical aptasensor, vascular endothelial growth factor, one-step, signal amplification, metallo nanoenzyme particles

## Abstract

In this study, a one-step electrochemical aptasensor was developed to detect the biomarker vascular endothelial growth factor (VEGF), an important protein in the pathogenesis of many retinal diseases, including age-related macular degeneration, diabetic retinopathy, retinopathy of prematurity, and retinal vein occlusion. The aptamer has a good affinity and can rapidly identify and capture VEGF based on its unique structure. We designed a VEGF aptasensor based on the aptamer recognition and complex metallo nanoenzyme particles as an electron exchange center and bridge between capture DNA and electrode. The aptamers maintained the hairpin structure to avoid nonspecific surface adsorption and expose the capture sequence outwards when the target was inexistent. Conversely, the aptamers opened the hairpin structure to release space to accomplish binding between VEGF and DNA, resulting in increased impedance. The performance of the electrochemical aptasensor is detected by electrochemical impedance spectroscopy (EIS). The limit of detection by EIS was as low as 8.2 pg ml^−1^, and the linear range was 10 pg ml^−1^–1 μg ml^−1^. The electrochemical aptasensor also showed high specificity and reproducibility.

## Introduction

Pathological changes due to exudation, bleeding, and hyperplasia caused by ocular neovascularization are a major cause of visual impairment. Vascular endothelial growth factor (VEGF) is key in ocular neovascularization and related to diseases such as retinal vein obstruction, diabetic retinopathy (DR), neovascular glaucoma, age-related macular degeneration (AMD), and retinopathy of prematurity (ROP). VEGF stimulates mitosis and migration of vascular endothelial cells, increasing the permeability of blood vessels (Lange et al., 2016; Zhao and Singh, 2018). Early treatment can significantly reduce complications and subsequent visual impairment caused by neovascularization. The difficulties associated with the treatment are related to early clinical diagnosis. Thus, it is necessary to establish an effective analytical system of VEGF, which is favorable for diagnosis, progression, anti-VEGF therapy dosage, and efficacy evaluation.

To date, several approaches for the detection of VEGF have been reported, including chemiluminescence (Long et al., 2018), fluorescence (Dang et al., 2019), photoelectricity (Da et al., 2018), colorimetry (Zhang et al., 2017), electrochemistry (Bozal-Palabiyik et al., 2019), and enzyme-linked immunosorbent assay (ELISA) (Chattaraj et al., 2016). However, most methods require tedious steps or large-scale equipment with a point of care use. The content of VEGF in the bodily fluid is as low as dozens to hundreds of pg ml^−1^. Hence, some methods are not sufficiently sensitive for application, while some enzyme-based methods are difficult to store. Among many new techniques, electrochemical sensors have received great attention in the detection of biomarkers owing to their characteristics of rapidness, sensitivity, cost-efficiency, point-of-care, and simple operation, rendering them suitable for clinical application (Ferapontova, 2018; Lima et al., 2018).

In recent years, aptamers that specifically bind to the target analyte are considered traditional substitutes of the antibody and are highly promising biorecognition elements (Jayasena, 1999; Ilgu and Nilsen-Hamilton, 2016). The dissociation constant of the aptamer and the target is 1 × 10^–12^–1×10^–9^ mol L^−1^. Thus, the affinity of the aptamers is similar to that of the antibodies (Jenison et al., 1994). The molecular weight of the aptamer is small. Therefore, the steric resistance to the target is smaller than that of the antibody to the antigen, which is conducive to the construction of the high-density complex. Aptamers are stable at room temperature and easily modifiable at the 5′ or 3′ end. Currently, aptamer-based electrochemical sensors have become a cutting-edge topic. They have been applied in sensing carcinoembryonic antigens (Gao et al., 2020), aflatoxin M1 (Stepanova et al., 2019), chloramphenicol (Zhu X et al., 2019), and thrombin (Wang et al., 2019).

In order to improve the sensitivity for the detection of biomarkers, substantial materials are used to construct the electrochemical sensors by increasing the specific surface area or accelerating electrical transmission. Enzymes are biological macromolecules with catalytic performance. They have the advantage of high efficiency and strong substrate specificity when applied in catalytic reactions. However, in the actual production process, natural enzymes are difficult to separate and purify, with high cost and poor stability, which brings much inconvenience to storage and use. Therefore, there is an urgent need for good stability and easy preparation of simulated enzymes to meet the needs of practical production and application. Nanoparticle-based catalysts have attracted increasing interest due to their simple synthesis, adjustable catalytic activity, high stability, low cost, and easy treatment (Kumar et al., 2017; Hu et al., 2018; Yao et al., 2018). Zhu et al. reported a CuS nanozyme with a large surface area to provide excellent catalytic activity for detecting dopamine and glucose (Zhu et al., 2019). Platinum (Pt) nanoparticle has been applied in a sensitive electrochemical immunosensor to detect thyroid-stimulating hormone down to 0.3 pg ml^−1^ (Nandhakumar et al., 2020). Nanozymes are promising alternatives to natural enzymes for electrochemical biosensors (Mahmudunnabi et al., 2020).

**SCHEME 1:**
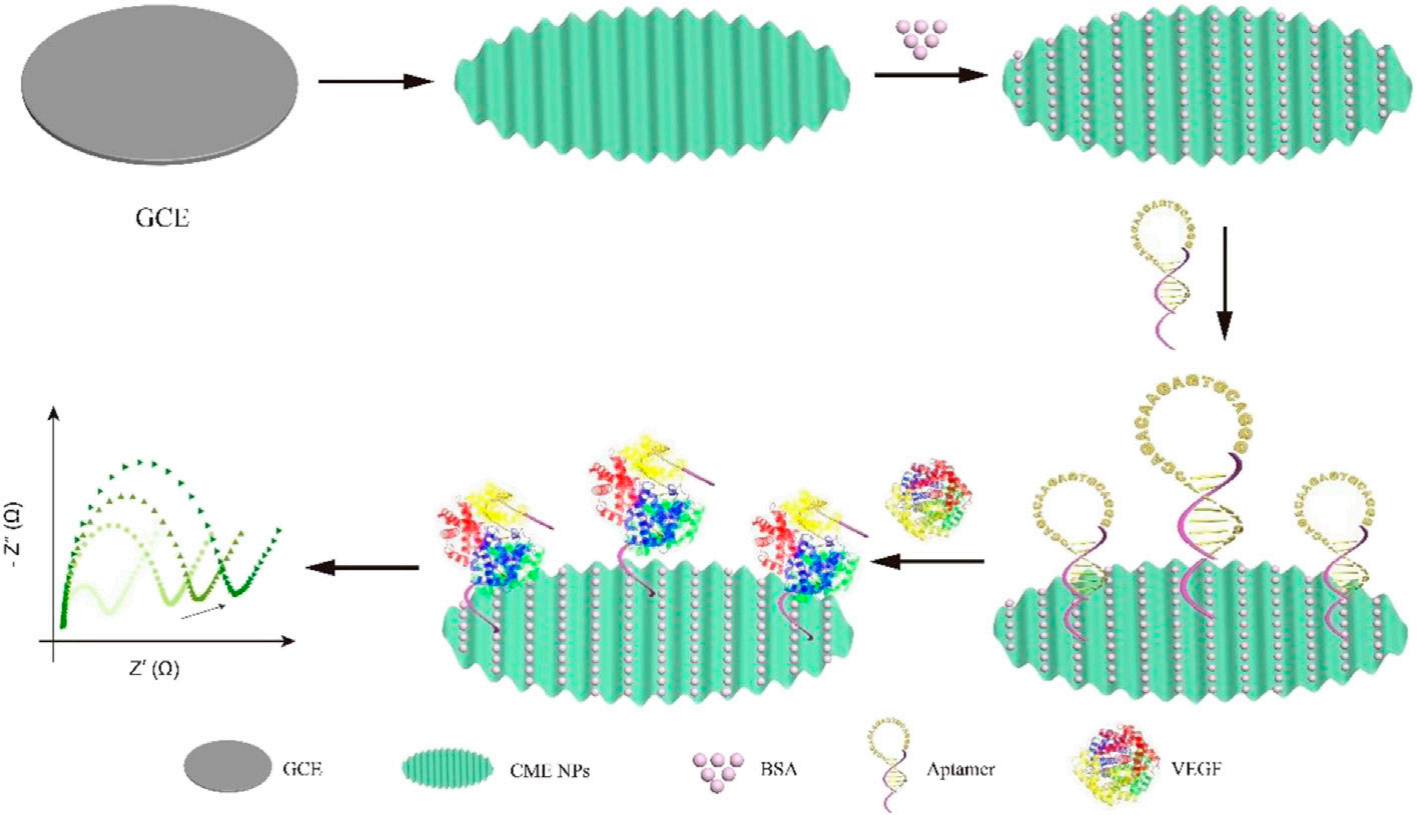
Schematic illustration of VEGF aptasensor based on CME NPs.

In order to develop a novel VEGF detection method for clinical use, we designed a simple, effective, and sensitive VEGF detective sensor based on aptamer recognition and (CME NPs) as an electron exchange center and bridge between capture DNA and electrode. The aptamers maintained the hairpin structure to avoid nonspecific surface adsorption and expose the capture sequence outwards when the target was inexistent. Conversely, the aptamers opened the hairpin structure to release the space to accomplish binding between VEGF and DNA, resulting in increased impedance. Thus, the impedance changes before and after target binding are exploited to realize the rapid and sensitive detection of VEGF (Scheme 1).

## Experimental Section

### Chemicals and Materials

Platinum acetylacetonate (Pt (acac)_2_, 98%), palladium acetylacetonate (Pd (acac)_2_, 99%) and bis(acetylacetonato)dioxomolybdenum (MoO_2_ (acac)_2_, 97%) were purchased from Shanghai Macklin Biochemical Co., Ltd. (China). Sodium iodide (NaI, AR), dimethyl formamide (DMF, AR), and polyvinyl pyrrolidone (PVP, MW = 30,000, AR) were purchased from Shanghai Aladdin Biochemical Co. Mucin, IL-6, IgG, VEGF A_165_, functionalized single-stranded DNA (ssDNA) with 5′ carboxylic acid groups, and sequence 5′-AAA​AAA​AAA​ACC​GTC​TTC​CAG​ACA​AGA​GTG​CAG​GGA​AAA​AGA​AGA​CGG-3′ were obtained from Sangon Biological Engineering Technology & Services Co., Ltd. (Shanghai, China). All reagents were of analytical grade and used as received without further purification. Milli-Q water was obtained using a Milli-Q water purification system (18.2 MΩ cm, Millipore, Molsheim, France).

### Instruments and Electrodes

The measurements of all electrochemical assays were performed on an AUTOLAB M204 electrochemical workstation (Metrohm Autolab B.V, Netherlands), connected to a computer data analysis system. The electrochemical impedance spectroscopy (EIS) experiments were carried out using a conventional three-electrode system consisting of a 3 mm glassy carbon working electrode (GCE), a platinum counter electrode, and an Ag/AgCl reference electrode. The XRD pattern of the obtained CME NPs was measured using a Smartlab 9 powder diffractometer with Cu K radiation (λ = 1.54 Å). TEM, HRTEM images, SAED pattern, and energy dispersive X-ray spectroscopy (EDX) mapping images were inspected using transmission electron microscopy (JEM2100F, Japan) with an accelerating voltage of 200 kV.

### Preparation of CME NPs

In a typical synthesis process (Mu et al., 2019) of CME NPs, 20 mg Pt (acac)_2_, 16 mg Pd (acac)_2_, and 16 mg MoO_2_ (acac)_2_ were firstly added into 10 ml DMF under stirring until a transparent solution was obtained. Then, 2 ml NaI solution (300 mg) was added. After stirring for 20 min, 160 mg PVP was added, and the mixed solution was stirred for another 20 min. The resulting solution was transferred into a Teflon-lined autoclave and sealed tightly. Then the autoclave was maintained at 150°C for 10 h. Subsequently, the mixture was transferred to a Teflon-lined stainless-steel autoclave and maintained at 150°C for 10 h. After cooling to room temperature naturally, the precipitate was collected and washed with ethanol several times. The final products were dried through lyophilization. As a control, Pt, Pd, and bimetal PtPd nanoparticles were synthesized through a similar process.

### Fabrication of Aptasensor

Before modification, a GCE was polished to a mirror using 0.05 μm alumina slurry, followed by thorough rinsing with ethanol and deionized water sequentially, sonication in deionized water for 1 min, drying by blowing N_2_ gas, and keeping it capped before use. Then, 5 μL CME NPs in chitosan solution was dropped on the GCE, and the electrode was dried in air for 2 h at room temperature. Next, the functionalized hairpin DNA was incubated in 20 mg ml^−1^ EDC (in 1.0 mM MES, pH 5.5) for 30 min with gentle shaking, followed by adding an equivalent volume of 20 mg ml^−1^ NHS for 5 min with gentle shaking. A volume of 5 μL mixture was dropped onto the modified GCE and kept capped at 4°C overnight. Then, the modified GCE was washed to remove unbound DNA, followed by incubation with 5% BSA for 1 h at room temperature to prevent nonspecific adsorption. Finally, the resulting GCE was washed with deionized water to remove redundant BSA and maintained at 4°C before use.

### Electrochemical Measurements

For the electrochemical immunoassay of the analyte, 5 μL of the standard VEGF solution or a blank sample was dripped onto the surface of the aptasensor to complete binding between the aptasensor and protein, followed by incubation for 1 h at room temperature. After rinsing with 10 mM phosphate-buffered saline (PBS), the EIS was recorded when the frequency range was 0.1–100 kHz, the amplitude of the applied sine wave potential was 10 mV, and at an open-circuit voltage in 0.1 M KCl solution containing 5 mM [Fe(CN)_6_]^3−/4−^. Subsequently, cyclic voltammetry (CV) was performed at scan potential −0.2 to −1.1 V at a sweep rate of 50 mV/s in 0.1 M KCl solution containing 5 mM [Fe(CN)_6_]^3^−^/4^−. The catalytic performance was recorded by CV using a potential window of −0.8 to +0.2 V at a sweep rate of 50 mV/s in PBS buffer containing 15 mM H_2_O_2_ under the protection of nitrogen.

## Results and Discussion

### Materials Characterization

**FIGURE 1:**
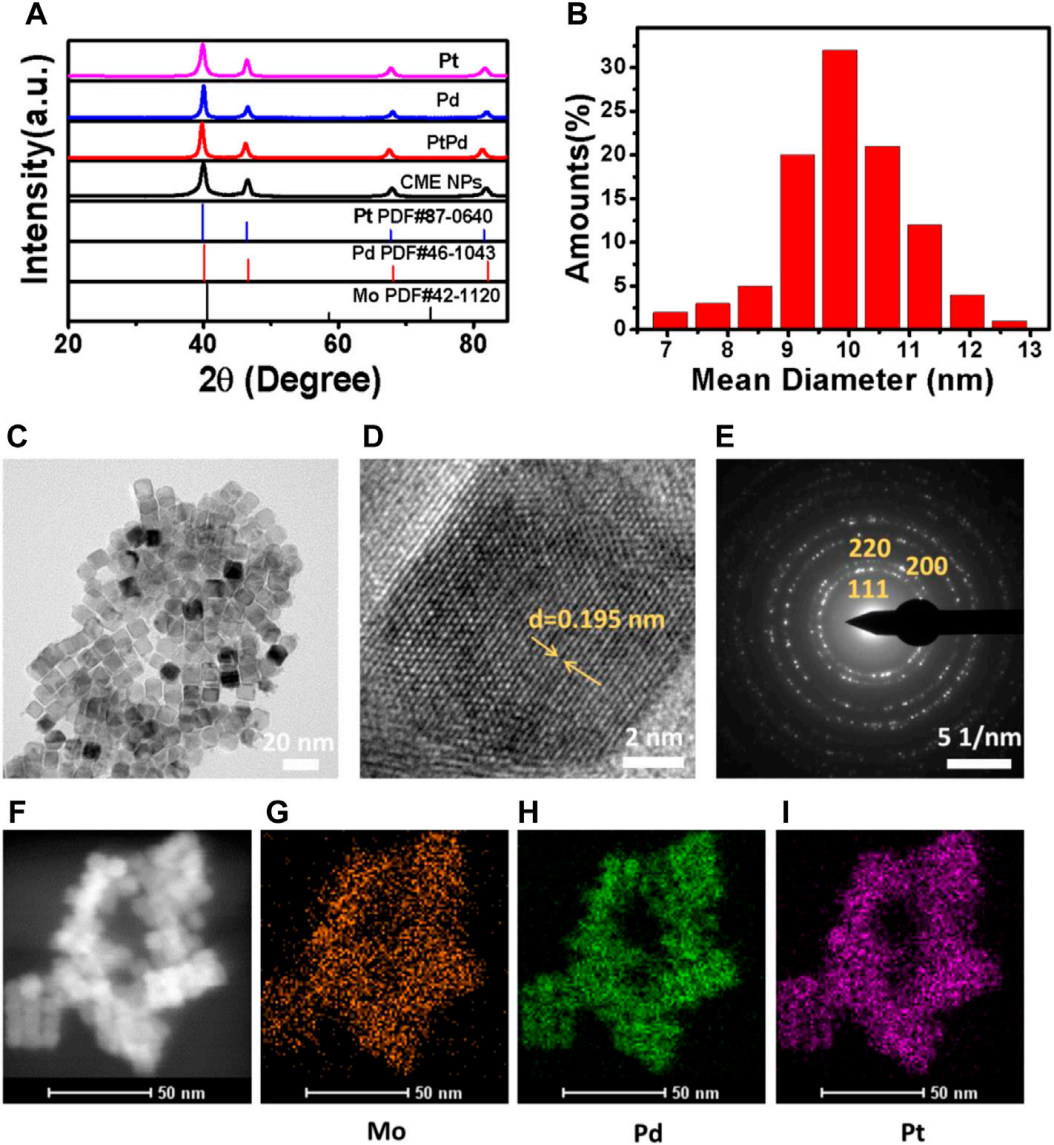
XRD **(A)**, size statistical analysis spectra **(B)**, TEM **(C)**, HRTEM **(D)**, SAED **(E)**, and TEM EDX mapping (Figures 1F–I) of the synthesized nanoparticles.

The XRD pattern of the synthesized CME NPs was firstly characterized. The results shown in Figure 1A indicate that all diffraction peaks were in good agreement with those of the cubic Pt in the JCPDS file PDF#87-0640, displaying the *fcc* structure of the CME NPs. No other diffraction peaks appeared in the spectra, indicating that doping of Pd (PDF#46-1043) and Mo (PDF#42-1120) has little effect on the final crystal structure. Similar diffraction peak locations of Pd, Mo, and Pt also played critical roles. All diffraction peaks agreed well with the report (Mu et al., 2019), indicating the successful synthesis of the product. The TEM image displayed in Figure 1C indicated that the obtained sample was composed of uniform cubic nanoparticles with a mean size of about 10 nm, similar to Pd, Pt, and PdPt (Supplementary Figures S2A–S2C). The size of the statistical analysis spectra shown in Figure 1B further confirmed the observation in Figure 1C. Clear lattice fringes with a d-spacing of 0.195 nm for the (200) plane shown in Figure 1D exhibited a perfect crystal structure of the obtained nanoparticles. The corresponding SAED (selected area electronic diffraction) pattern shown in Figure 1E further confirmed the crystal structure of the sample. From TEM EDX mapping (Figures 1F–I), it can be observed that Pt, Pd, and Mo, three elements, covered the sample uniformly, indicating the successful and uniform combination of the three kinds of elements. The energy dispersive X-ray spectrum shown in Supplementary Figure S1 further confirmed the existence of Pt, Pd, and Mo in the sample (C and Cu coming from the grid). The valence state variation of the CME NPs was studied using XPS (Supplementary Figures S2D–S2F). XPS spectra show Pt 4f, Pd 3d, and Mo 3d, the Pt species on the surface of the CME NPs which are mainly in the form of Pt^0^ 4f_5/2_ (73.941eV), Pt^0^ 4f _7/2_ (70.458 eV), Pt^2+^ 4f _5/2_ (74.205eV), Pt^2+^ 4f _7/2_ (70.94eV), the Pd species of Pd^0^ 3d_3/2_ (340.8 eV), Pd^0^ 3d_5/2_ (334.5, 334.9, 335.2 eV), Pd^2+^ 3d_5/2_ (340 eV), the Mo species of Mo^4+^ 3d_3/2_ (234, 235.3 eV), Mo^5+^ 3d_3/2_ (235 eV), Mo^5+^ 3d_5/2_ (230.7 eV), Mo^6+^ 3d _3/2_ (232 eV).

### Electrodes Electrochemical Characteristics

**FIGURE 2:**
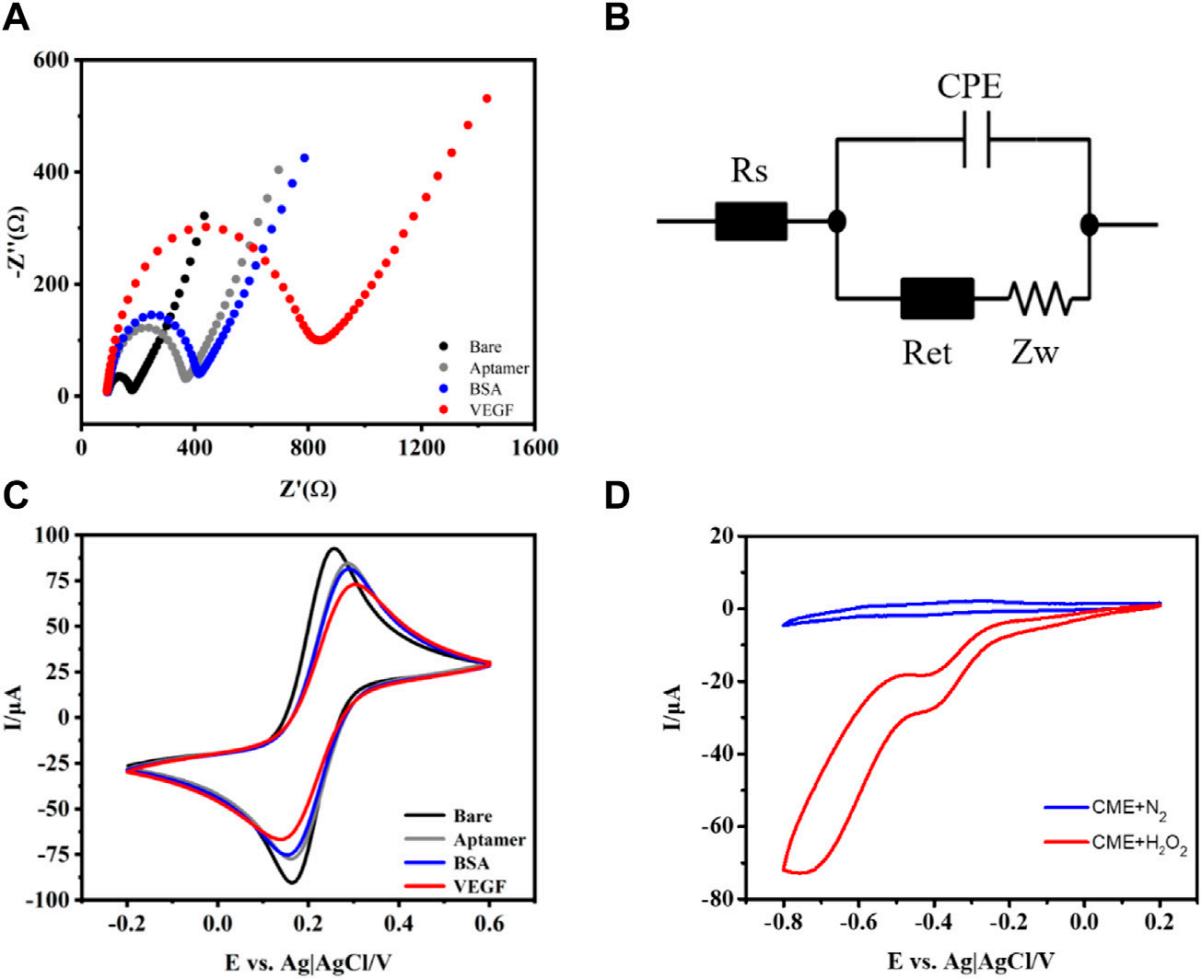
Nyquist plots of the EIS **(A)**, equivalent circuit diagram **(B)**, CV for different modified electrodes **(C)**, and in PBS buffer with or without H_2_O_2_ **(D)**.

In order to monitor aptasensor fabrication, the CV and EIS in 0.1 M PBS (pH 7.4) containing 5 mM [Fe(CN)_6_]^3/4−^ were recorded. As shown in Figure 2A, the bare GCE exhibits a very low charge transfer resistance according to the equivalent circuit diagram (Figure 2B). The Randles equivalent circuit possesses several parameters, including solution resistance (Rs), constant phase element (CPE), and interfacial electron transfer resistance (Ret), equal to the semicircle diameter of EIS and the Warburg impedance (Zw). When aptamer/CME NPs were modified on the electrode surface, the Ret value increased. This is probably attributed to the electrostatic repulsive force from single-stranded aptamer with negative charges on its phosphate backbone and [Fe(CN)_6_]^3/4−^ solution. Subsequently, the Ret value of BSA/aptamer/CME NPs further increased a little bit because the formed layer acted as a mass-transfer blocking barrier (Cheng et al., 2012). Finally, a significant increase in Ret was observed when VEGF (10 ng ml^−1^) was bound to aptamers because the aptasensor was introduced with a mass-transfer blocking barrier from VEGF. The increased Ret indicated that the electrochemical was established successfully. Meanwhile, the EIS of the pure chitosan-modified group did not change significantly when the same concentration of VEGF was added (Supplementary Figure S3). Based on the above experiment, the CME NPs played the role of a bridge between capturing DNA and the electrode and enhanced signal transmission. As shown in Figure 2C, the changes in corresponding CVs of the aptasensor demonstrate a trend that agrees with the EIS experimental results. As shown in Figure 2C, the changes in corresponding CVs of the aptasensor demonstrate a trend that agrees with the EIS experimental results. The peak current decreased clearly after aptamer assembly on the electrode surface, indicating that assembled aptamers hinder the diffusion of ferricyanide toward the electrode surface. Subsequently, when BSA was introduced to block nonspecific adsorption sites on the electrode surface, the peak current decreased further due to the steric hindrance effect of BSA. After incubating the aptasensor in VEGF, a decrease in current was observed, indicating that the aptasensor was successfully assembled. The CME NPs were dropped on a GCE, compared with N_2_-saturated in the PBS buffer, and the presence of H_2_O_2_ in the electrolyte makes a sharp stripping peak in Figure 2D. Meanwhile, bare GCE in H_2_O_2_ solution shows a relatively small response (Supplementary Figure S4), indicating that CME NPs possess the catalytic activities for H_2_O_2_. As shown in Figure 2D, the bare GCE exhibits an unconspicuous change of peak in PBS (pH 7.4, 10 mM) containing 15 mM H_2_O_2_ under the protection of nitrogen. In contrast, when CME NPs were modified on the electrode surface, the current reduction response increased significantly, which shows that CME NPs possess excellent catalytic activity for H_2_O_2_. As shown in Supplementary Figure S4, the Pd and Pt modified electrodes both demonstrated relatively small activity. Although the PtPd modified electrodes showed high activity, the multiples of change were not as good as the CME NPs modified electrode. Therefore, the CME NPs have the most potential under the above-mentioned conditions.

### Optimization of the Aptasensor

**FIGURE 3:**
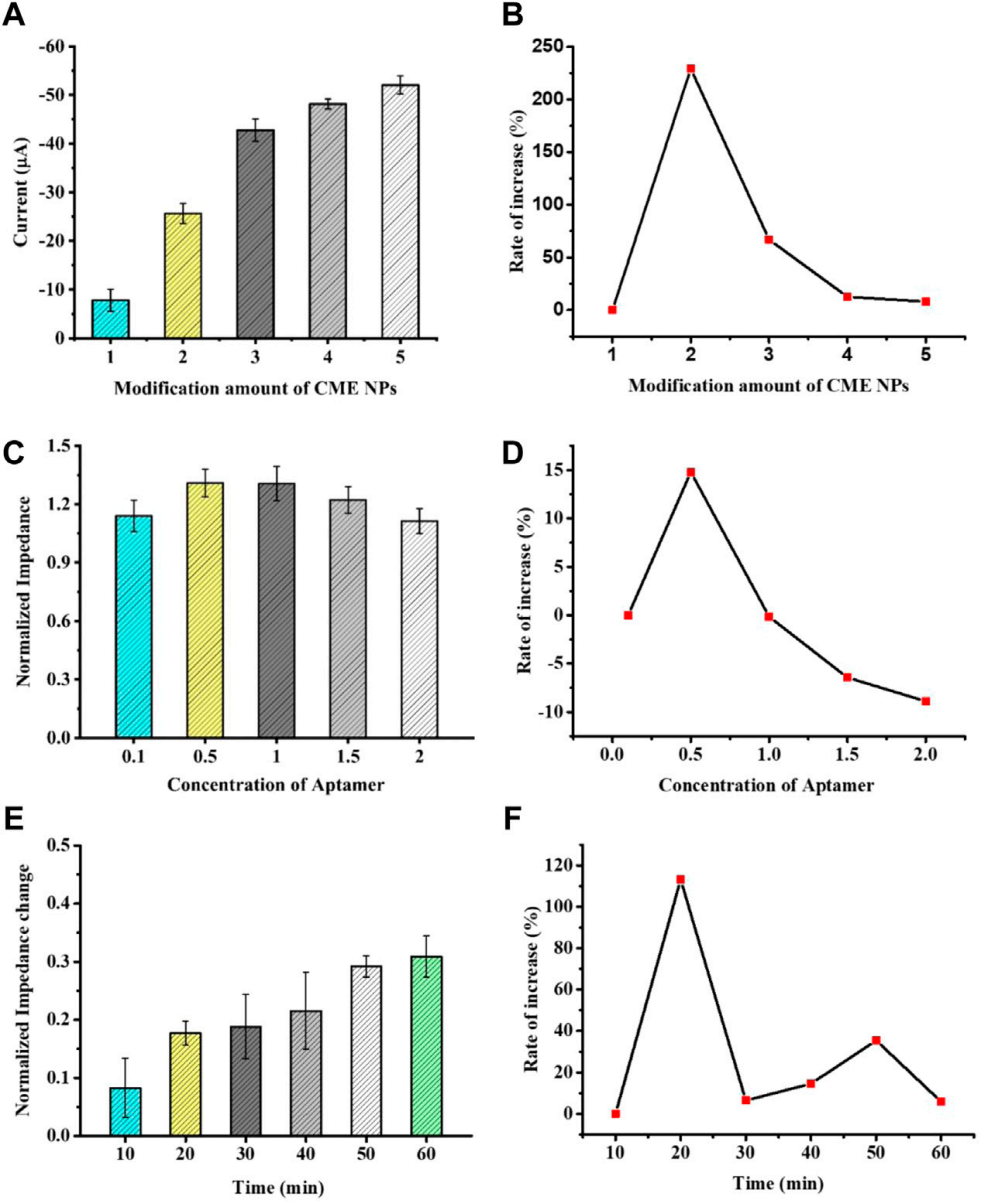
The optimization of experimental conditions with modification amount of CME NPs **(A)**, concentration of aptamer **(C)**, and time of hybridization **(E)**. The rate of increase under certain conditions with CME NPs **(B)**, aptamer **(D),** and time of hybridization **(F)**. Error bars, SD, *n* = 3.

In order to display the best catalytic performance, as shown in Figures 3A, B, the peak current rapidly increased with the increase in CME NPs from 1.0 mg ml^−1^ to 3.0 mg ml^−1^ and slowly increased with the increase in CME NPs from 3.0 mg ml^−1^ to 5.0 mg ml^−1^. The above experimental results possibly put down to thick coating blocking the electrons transfer (Zhang et al., 2020). Besides, the electrochemical stability of the aptasensor was reduced with the increase in CME NPs (Supplementary Figure S5). Thus, the optimized modification amount of CME NPs was determined as 3.0 mg ml^−1^. The parameter of each concentration was measured three times on different electrodes.

In order to investigate the effect of the aptamer concentration on sensor performance, the electrode modified with CME NPs was treated with 0.1, 0.5, 1.0, 1.5, and 2.0 M aptamer DNA and the same concentration of VEGF was dropped on it. When the electrode surface modified with the aptamer was used to detect the VEGF, the impedance of the system was increased. The increase in Ret suggests that the binding between the biomarker and the aptamer leads to a decrease in the mass-transfer efficiency and alters the dielectric and conductive properties of the electrode surface (Roushani et al., 2019; Mahmoud et al., 2020). Furthermore, to confirm the effect of the aptamer concentration on sensor performance, the normalized impedance (Ra/Rb, Ra represents the Ret of the electrode after treating with VEGF or control solution and Rb represents the Ret of the electrode before treating with VEGF or control solution) was plotted as the ordinate and the concentration of the aptamer was taken as the abscissa. The normalized impedance allows each electrode to serve as its own internal control and reduces individual differences of electrodes. As seen in Figures 3C, D, as the aptamer concentration increases, the normalized impedance shows a trend of increasing first and then decreasing. This phenomenon could be attributed to a modest increase in the aptamer bringing extra bonding capacity and an excess increase in the aptamer bringing steric hindrance. Thus, 1.0 M was selected in the subsequent experiment. The parameter of each concentration was measured three times on different electrodes.

Incubation time is also an important factor affecting the analysis performance of the aptasensor. To investigate the best incubation time between aptamers and proteins, the modified GCEs were incubated at the same concentration of VEGF at 37°C, and data were collected at different time points. As shown in Figures 3E, F, the normalized impedance increased with an increase in incubation time. When the incubation time reached 60 min, the impedance reached a response plateau, indicating that the immune binding in the reaction was saturated. Therefore, we selected 60 min as the aptasensor analysis incubation time.

### Analytical Performance

**FIGURE 4:**
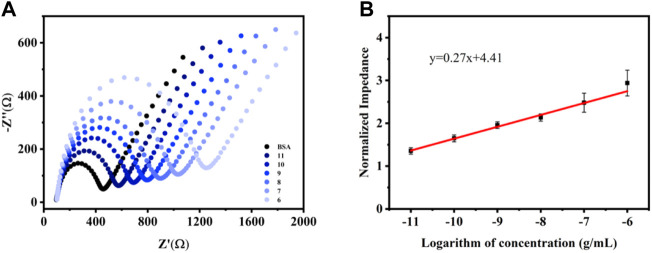
EIS responses **(A)** and the corresponding calibration plot of normalized impedance **(B)** *vs.* log VEGF in 0.1 M KCl solution containing 5.0 mM [Fe(CN)_6_]^3−/4−^. Error bars, SD, *n* = 3.

Under the optimized experimental conditions, the aptasensor was modified to 5 μL 3.0 mg ml^−1^ CME NPs, and the concentration of the aptamer was 1.0 M. Then, a 5 μL of the diluted, different VEGF concentrations solution was added to the modified electrode surface and incubated for 60 min to ensure that the liquid completely covered the GCE. Subsequently, the unbound proteins were washed with 1 ml of deionized water and characterized by EIS. In the EIS measurements, the Ret signal increased according to the binding of VEGF to the aptamer (Figure 4A). The trend of increased Ret signal slows down upon reaching a certain concentration because the quantitative aptamer has a threshold value to capture VEGF. The working curve revealed that the response range of the sensor is 10 pg ml^−1^–1 μg ml^−1^ (VEGF concentration), as suggested by the correlation between Ret and the logarithm of the analyte concentration (Figure 4B) (*y* = 0.27x + 4.41, *R* ^2^ = 0.99). The detection of limit (LOD) was acquired from 3SD corresponding to the 10 blank tests and calculated as 8.2 pg ml^−1^.

### Specificity

**FIGURE 5:**
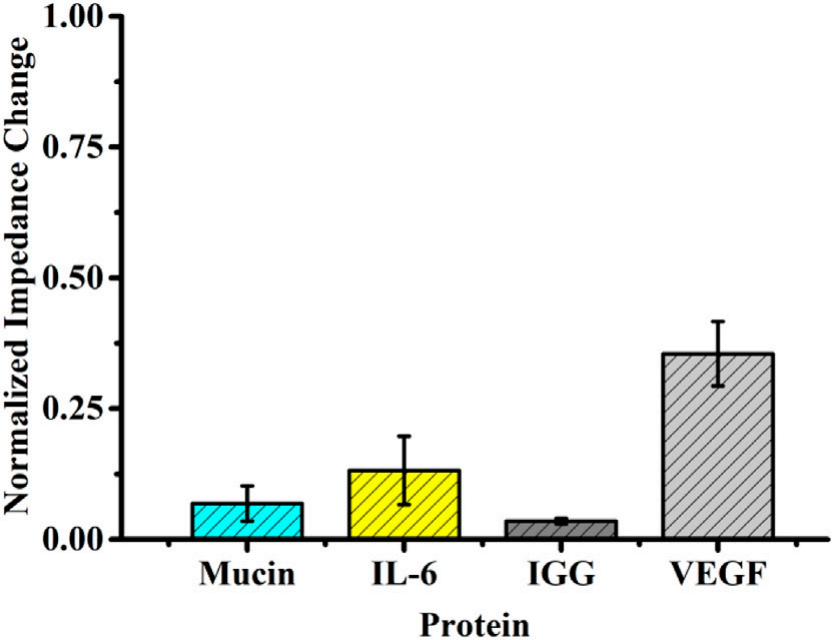
Selectivity evaluation of the developed method for detecting VEGF (10 pg ml^−1^) against other proteins (100 pg ml^−1^).

In order to explore specificity, the self-assembled aptasensor was incubated in the presence of an interfering protein (negative control), that is, the interfering protein replaced VEGF as the captured target protein by setting the concentration of the interfering protein to be 10-fold of that of VEGF, analyzed by EIS. The normalized impedance change was selected as the standard of judgment. Although the normalized impedance change value of the interfering protein binding to the sensor surface was significantly increased, it was still far from the normalized impedance change value caused by the corresponding concentration of VEGF (Figure 5).

### Application

In order to evaluate the application prospect of the aptasensor in detecting VEGF, referring to the previously established working graph, we used the aptasensor to detect 100 pg ml^−1^ VEGF in 1％ fetal bovine serum, and its recovery was 106％–115％.

Patients with DR had high levels of VEGF in their tears, which were correlated with the severity of the DR. The concentration of VEGF in DR patients’ tears was 1048.8 ± 194.2 pg ml^−1^, according to our previous work (Mei et al., 2021). 10 μL of tear samples was collected and diluted 10 times as control to counteract background interference. In the experimental group, exogenous VEGF was added to 100 pg ml^−1^ in diluted tears. Recovery was calculated between 96% and 112%.

## Conclusion

The electrochemical biosensors are mainly divided into aptasensor and immunosensor by recognition (Table 1). This method, based on the aptamer recognition and CME NPs as an electron exchange center and bridge between capture DNA and electrode, which was non-enzyme and without labels, does not involve complex primer design and detection of VEGF and range of 10 pg ml^−1^–1 μg ml^−1^. This method is simple, convenient to operate, economical, time-saving, and simple to implement. Moreover, it has a reference value for establishing detection methods for other proteins or target objects, including VEGF.

**TABLE 1:** Comparison of the electrochemical biosensors.

Method	Materials	Recognition	LODs	Linear range	Targets	Ref
Impedimetric	AuNCs/IL	Aptamer	6.7 pM	2.5–250 pM	VEGF_165_	Shamsipur et al. (2015)
Impedimetric	Carbon nanohorns/AuNPs	Aptamer	0.5 pg ml^−1^	1–1000 pg ml^−1^	CBZ	Zhu C et al. (2019)
Impedimetric	-	Anti-PSA	640 pg ml^−1^	640 pg ml^−1^–62.5 ng ml^−1^	PSA	Diaz-Fernandez et al. (2021)
Impedimetric	AuNPs	Anti-CRH	2.7 ug mL^−1^	10.0–80 ug mL^−1^	CRH	Duran et al. (2019)
Impedimetric	G/TiO_2_	EDIII	2.81 ng ml^−1^	62.5–2000 ng ml^−1^	DENV	Siew et al. (2021)
Impedimetric	PEDOT/Au NP	Anti-VEGF	0.5 pg ml^−1^	1–20 pg ml^−1^	VEGF_165_	Kim et al. (2019)
Voltammetric	Graphene oxide/ssDNA/PLLA NPs	Anti-VEGF	50 pg ml^−1^	0.05–100 ng ml^−1^	VEGF_165_	Pan et al. (2017)
Impedimetric	PdPtMo CME NPs	Aptamer	8.2 pg ml^−1^	10 pg ml^−1^–1 μg ml^−1^	VEGF_165_	This work

## Data Availability

The original contributions presented in the study are included in the article/Supplementary Material. Further inquiries can be directed to the corresponding authors.
