# Supplementary material for: A One-Step Electrochemical Aptasensor Based on Signal Amplification of Metallo Nanoenzyme Particles for Vascular Endothelial Growth Factor
Source: Front Bioeng Biotechnol. 2022 May 9;10:850412. doi: 10.3389/fbioe.2022.850412 (PMC9124786; doi:10.3389/fbioe.2022.850412)
Supplement: Supplementary file 1 [file DataSheet1.DOCX]

Supporting information

Figure S1. Energy dispersive X-ray spectrum of the synthesized nanoparticles.
